# Carnitine palmitoyl transferase-1A (CPT1A): a new tumor specific target in human breast cancer

**DOI:** 10.18632/oncotarget.6964

**Published:** 2016-01-21

**Authors:** Sabina Pucci, Maria Josè Zonetti, Tommaso Fisco, Chiara Polidoro, Gianfranco Bocchinfuso, Antonio Palleschi, Giuseppe Novelli, Luigi G. Spagnoli, Paola Mazzarelli

**Affiliations:** ^1^ Department of Biomedicine and Prevention, Tor Vergata University of Rome, Rome, Italy; ^2^ Department of Chemical Sciences and Technologies, Tor Vergata University of Rome, Rome, Italy

**Keywords:** breast cancer, carnitine palmitoyl transferase-1A, HDAC inhibitor, tumor metabolism, tumor specific target

## Abstract

Transcriptional mechanisms epigenetically-regulated in tumoral tissues point out new targets for anti-cancer therapies. Carnitine palmitoyl transferase I (CPT1) is the rate-limiting enzyme in the transport of long-chain fatty acids for β-oxidation. Here we identified the tumor specific nuclear CPT1A as a product of the transcript variant 2, that doesn't retain the classical transferase activity and is strongly involved in the epigenetic regulation of cancer pro-survival, cell death escaping and tumor invasion pathways. The knockdown of CPT1A variant 2 by small interfering RNAs (siRNAs), was sufficient to induce apoptosis in MCF-7, SK-BR3 and MDA-MB-231 breast cancer cells. The cell death triggered by CPT1A silencing correlated with reduction of HDAC activity and histone hyperacetylation. Docking experiments and molecular dynamics simulations confirmed an high binding affinity of the variant 2 for HDAC1. The CPT1A silenced cells showed an up-regulated transcription of pro-apoptotic genes (BAD, CASP9, COL18A1) and down-modulation of invasion and metastasis related-genes (TIMP-1, PDGF-A, SERPINB2). These findings provide evidence of the CPT1 variant 2 involvement in breast cancer survival, cell death escape and invasion. Thus, we propose nuclear CPT1A as a striking tumor specific target for anticancer therapeutics, more selective and effective as compared with the well-known HDAC inhibitors.

## INTRODUCTION

Carnitine Palmitoyl transferase 1 (CPT1) physiologically resides at the outer mitochondrial membrane and is a site for intracellular regulation of lipid metabolism, transporting long-chain fatty acids into mitochondria for β-oxidation (together with CPT2 and Carnitine/acyl-Carnitine Translocase) [1, 2]. Three different CPT1 isozymes are identified: CPT1A (also called L-CPT1), CPT1B (called M-CPT1) and the recently described CPT1-C. While CPT1B is expressed only in brown adipose tissue, muscle and heart and CPT1C in the endoplasmic reticulum of neurons [3], CPT1A has a more widespread distribution and it is found in liver, pancreas, kidney, brain, blood and embryonic tissues [1, 2]. CPT1A displays higher affinity for its substrate carnitine and a lower affinity for the physiological inhibitor malonyl-CoA compared to the CPT1B muscle isoform [4]. Recently, we demonstrated that CPT1A was significantly down-regulated in the cytosolic compartment of human colorectal and breast cancer tissues and in several neoplastic cell lines, whereas it showed a peculiar localization in the nuclei of the same samples [5].

We reported the existence of a correlation among nuclear CPT1A, histone acetylation level and histone deacetylase (HDAC) activity in tumoral tissues and neoplastic cell lines. In particular, we displayed that HDAC-1 protein co-precipitated with nuclear CPT1A [5]. HDAC multiprotein complexes are recruited to specific genomic sites by regulatory proteins [6]. It has been shown that recruitment of HDAC by a transcription factor complex results in tightening of chromatin structure, thus the transcriptional machinery is prevented from accessing the DNA. The transcriptional repression of specific genes, induced by HDAC recruitment, could be involved in cancer development [7]. Various types of cancer exhibit aberrant HDAC level and/or sustained activity. Thus, HDACs inhibition represents a new strategy in human cancer therapy [8, 9, 10]. A wide variety of HDAC inhibitors (HDACI) of both natural and synthetic origin has been reported and some of them are currently under phase I or phase II clinical trials [11, 12]. They act by arresting cell cycle progression and induce differentiation and apoptotic death in tumor cells, even so, the knowledge on drug specificity is still limited and gene targets await to be defined. In the previous report, we hypothesized that CPT1A could function by favoring, in some way, the HDAC-mediated deacetylation of specific genes, with the effect of a selective suppression of genes involved in the control of cell cycle/growth [7, 13, 14].

At the purpose to better define the function of CPT1A in the epigenetic regulation of cancer survival, in the present report we use MCF-7 cell line as *in vitro* model of human breast cancer. In these cells, we had previously detected a CPT1A mRNA transcript splice variant, termed variant 2 (CPT1Av2) (NM_001031847), that was undetectable in the corresponding non neoplastic MCF12F cell line. This transcript variant codifies for a protein which differs in only 11 aminoacids from CPT1A variant 1 (CPT1Av1), at the C-terminus. Here, we firstly identify the cellular localization of the transcript variant 2 product only in the nucleus of tumoral cells. The nuclear CPT1A has no classical transferase activity, as compared with the variant 1. Hence, we used small interfering RNA (siRNA) sequences against the transcript variant 2 alone and against both mRNA variants of CPT1A. The siRNA targeting of variant 2 CPT1A induced: i) a significant decrease of HDAC activity, ii) a significant increase of histone acetylation level, iii) apoptotic cell death. Oligogene arrays demonstrated that in variant 2-siRNA transfected MCF-7 cells, proapoptotic factors such as BAD, CASP9 etc. were significantly up-regulated, whereas metastasis and invasion-related genes (TIMP-1, SERPINB2, PDGF-A, etc.) were down-modulated. Furthermore, the interaction among the two isoforms of CPT1A and HDAC1 has been characterized by homology molecular models, docking experiments and molecular dynamics simulations, confirming an higher affinity of the variant 2 for HDAC1 in respect to the variant 1.

In conclusion, CPT1Av2, expressed in the nuclear compartment of breast cancer cells, interacts with HDAC1 molecule, contributing to epigenetic regulation of genes involved in cancer-relevant cell death and invasion pathways. Results obtained by gene silencing firmly delineate CPT1A as an intriguing target for more selective anti-neoplastic therapies.

## RESULTS

### Nuclear CPT1A variant 2 does not show classical transferase activity

**Figure 1:**
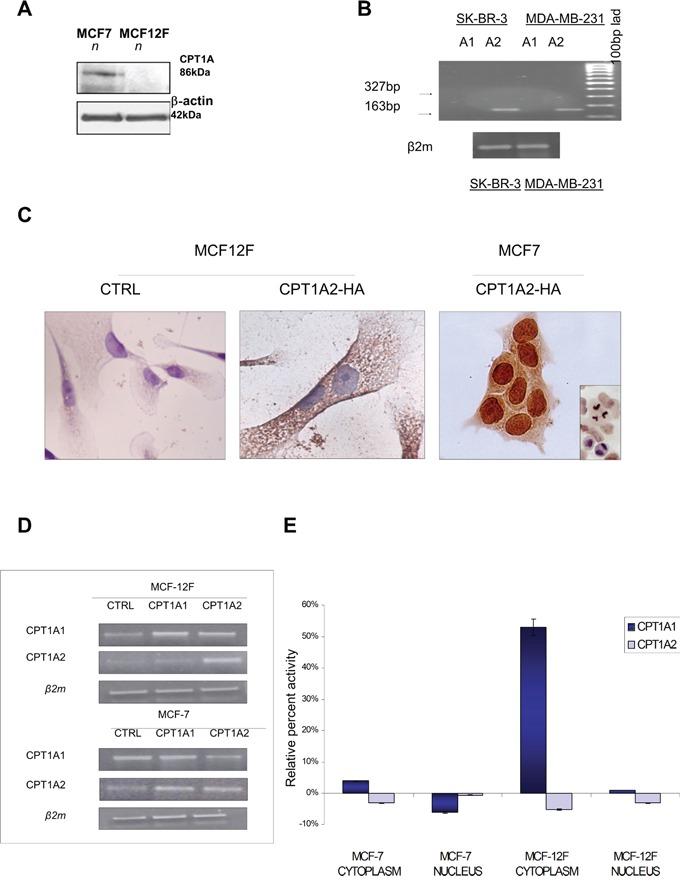
Protein expression, localization and transferase activity of CPT1A in MCF7 breast cancer cells compared to MCF12F control cells **A.** Western blot analysis of CPT1A from nuclear extracts of MCF7 and MCF12F cells. β-actin protein level was shown as normalizer. **B.** RT-PCR analysis of CPT1A isoforms (CPT1Av1 and CPT1Av2) expression in SK-BR-3 and MDA-MB-231 cells. 327bp was the expected size for the variant 1 amplicon, the classical form of CPTI-A. As shown only the CPTI-Av2 was detected in SK-BR3 and MDA-MB-231 cells (amplicon size: 163 bp). **C.** Immunostaining of HA was performed in MCF-7 and MCF12F cells transfected with empty plasmid (CTRL) or CPT1Av2. **D.** Transfection efficiency was evaluated by RT-PCR after 72 hrs. Amplification for β2-microglobulin was performed as control **E.** Spectrophotometrical assay was performed to analyze CPT activity from nuclear and cytoplasm extracts of MCF-7 and MCF12F transfected cells. Histograms show the relative percent activity obtained by Ellman's reagent at 412 ηm, from three independent experiments.

A CPT1A mRNA transcript splice variant, termed variant 2 (CPT1AV2) (NM_001031847) has been previously identified in the MCF-7 cell line [5]. This transcript variant codifies for a protein (NP_001027017) which is 17 aminoacids shorter than CPT1A variant 1 (CPT1Av1) (NP_001867.2), at the C-terminus. Western blot analysis of nuclear extracts from MCF7 cancer cells and MCF12F cells, derived from normal mammary gland, confirmed the presence of CPT1A (86kDa) only in the nuclei of breast cancer cells line (Figure 1A). In order to validate the presence of this peculiar transcript in breast cancer cells with different features and aggressiveness, the expression of CPT1A variant 1 and variant 2 were evaluated, by RT-PCR, also in cell lines representing other breast cancer phenotypes, MDA-MB-231 and SK-BR-3, the former derived from a basal phenotype and the latter luminal B PR/Her2+ expressing breast cancer cells. Unexpectedly, as shown in Figure 1B, only the expression of CPT1Av2 was observed in these two cell lines, the presence of the classical form CPTA 1Av1 was completely lost (Figure 1B).

In order to better define the subcellular localization of the CPT1Av2, and the role of this splice variant transfection experiments were performed in MCF7 and MCF12F cells. The CPT1Av2 sequence was cloned in HA expressing vector, as described in material and methods. Transfection efficiency was evaluated by RT-PCR after 72 hrs (Figure 1D), and immunocytochemistry was performed on transfected cells with anti-HA antibody to identify the CPT1Av2 localization (Figure 1C). A strong proliferative advantage was observed after 48 and 72 hours only in MCF7 CPT1Av2 transfected cells (data not shown). In 85% MCF7 transfected cells was observed a strong nuclear localization. On the contrary, in MCF12F the anti-HA antibody recognized the transfection products only in the cytoplasm, as shown in Figure 1C. These results strongly indicate that the localization and function CPT1A variant 2(CPT1Av2) were conditioned by the tumoral intracellular *milieu* that drives it to the nuclear context.

In order to characterize the function of the nuclear CPT1A, transferase activity assay was performed in MCF7 and MCF12F cells transfected with the CPT1Av1 or CPT1Av2 constructs. In MCF7 and in MCF12F cells transfected with CPT1Av2 the transferase assay did not exhibit increased enzymatic activity. Conversely, cells transfected with classical variant 1 showed an increased transferase activity, more significant in the MCF-12F cells, as compared to MCF7 (Figure 1E).

### Variant 2 siRNAs specifically silenced nuclear CPT1A expression

**Figure 2:**
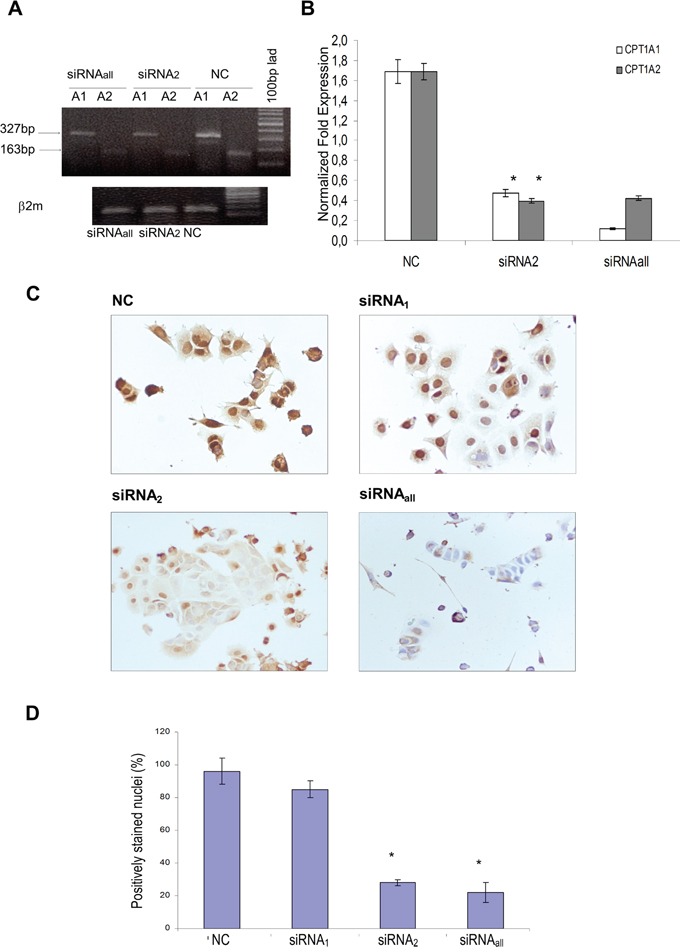
Expression levels of the two CPT1A isoforms (CPT1Av1 and CPT1Av2) in 48h silenced MCF-7 **A.** Cells were transfected with siRNA matching both the two variants (siRNAall), the transcript variant 2 (siRNA_2_) or scrambled sequence (NC). β2-microglobulin house-keeping gene was amplified to normalize template input. Results are representative of three independent experiments. **B.** Real time RT-PCR analysis. Data are expressed after normalizing to β2-microglobulin levels (endogenous control). Results are mean values (± standard deviations) of triplicate from two independent experiments. *P value < 0.01. **C.** CPT1A immunostaining in MCF-7 cells 48h after silencing with siRNAs matching the variant 1 (siRNA_1_), the variant 2 (siRNA_2_) or targeting both the two CPT1A variants (siRNA_all_). Images are representative of three independent experiments. **D.** A minimum of 100 cells per sample were observed using optical microscope at 20X magnification lens. The results (means values ± standard deviations) were given as percentage of CPT1A positively-stained nuclei per total cell nuclei number. *P= 0.03, siRNA_2_ or siRNA_all_-transfected *versus* mock transfected (NC).

Small interfering RNAs (siRNAs), matching the CPT1Av1 and CPT1Av2 were designed and used to transiently transfect neoplastic MCF-7 cells. We obtained efficient CPT1A down-regulation by transfection with siRNAs targeting the CPT1Av1 or CPT1Av2 alone (siRNA_1_, siRNA_2_) or both the transcript variants (siRNA_all_) for 48h and 72h of treatment. The efficiency of silencing was evaluated both at mRNA (Figure 2A, 2B) and protein level (Figure 2C, 2D) by Real time RT-PCR and immunocytochemistry. The transfection with siRNA_2_ reduced levels of both the CPT1A mRNA variants, suggesting the existence of a regulatory loop in the differential splice variants formation (Figure 2B). However, RNA interference differentially affected the two CPT1A isoforms at the protein level in the cytoplasmic and nuclear cell compartments. In fact, as shown by immunocytochemistry (Figure 2C), siRNA_2_ treatment of MCF-7 cells strongly decreased only the nuclear CPTIA staining (Figure 2C, 2D). In contrast, siRNA_1_ treatment did not affect nuclear CPTIA expression, and the full CPT1A gene silencing (siRNA_all_) knocked down the protein both from the cytoplasm and nucleus (Figure 2C).

### CPT1A2 gene silencing induces apoptosis in breast cancer MCF-7 cells

To evaluate the effects of CPT1A silencing on viability and death of tumoral cells, we assayed apoptosis induction inCPT1Av2 silenced and/or butyrate treated MCF-7 cells. Human MCF12F non cancerous mammary cells were used as control. Light microscopy revealed poor morphology and an higher amount of detached cells in the siRNA_2_ treated MCF-7 cells, compared with the mock-transfected ones.

**Figure 3:**
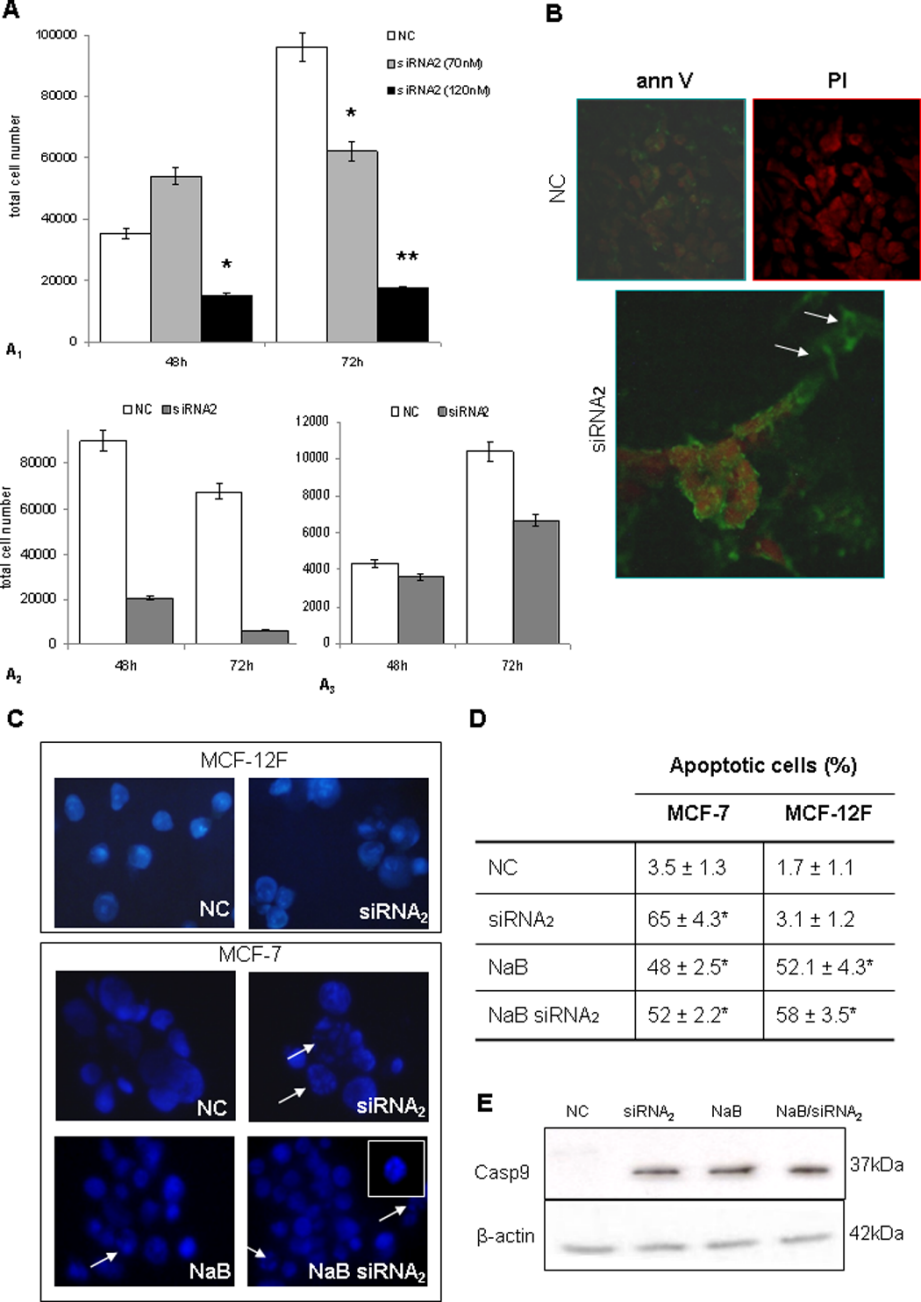
**A.** Cell viability after CPT1Av2 silencing in MCF-7 (*A1*), SK-BR-3 (*A2*) and MDA-MB-231 (*A3*) cells. Dye exclusion test (trypan blue staining) performed in cells transfected for 72h with siRNA2 at different concentrations. Decreased viability was significant at 70 nM CPT1A2 siRNAs. The cell viability on SK-BR-3 and MDA-MB-231 and the apoptotic assay were performed at 70 nM siRNAs concentration. Results are mean values ± standard deviations of three independent experiments. *P= 0.05, siRNA2 *versus* NC. **P<0.001, siRNA2 *versus* NC. **B.** Apoptosis detection with Annexin V (FITC) and propidium iodide (red) staining (40X magn.) The cells seeded in 4 wells/chamberslides were transfected for 72h with scrambled sequence (NC) or CPT1A siRNA2. Silenced cells showed early (Ann+, PI-) and late (Ann+, PI+) apoptotic events (arrows), as compared with un-treated cells. **C.** HOECHST DNA staining. Images show representative results of three independent experiments. Arrows indicates apoptotic figures and nuclear fragmentation. 5mM sodium butyrate was added in culture 48h after silencing (72h full incubation time), or alone for 24h. A particular of a nucleus is shown in the upper right side. Original magnification 100X. **D.** The results of HOECHST staining obtained from three independent experiments are given as percentage of apoptotic figures per total cell nuclei number. Asterisks indicates statistical significant differences (*P*≤0.05) in treated cells *versus* scrambled control (NC). Butyrate alone was able to induce apoptotic cell death also in MCF-12F control cells. **E.** CASPASE-9 expression in protein extracts from CPT1Av2 silenced (siRNA_2_) or/and treated with 5mM sodium-butyrate (NaB) MCF-7 cells *versus* scrambled control (NC).

The count of viable cells by dye exclusion test showed a significant decrease of MCF-7 cells 72h after siRNA transfection, as compared with mock-transfected cells (NT) (Figure 3A_1_).

siRNA transfections were performed also in MDA-MB-231 and SK-BR-3 breast cancer cells in order to define the role of CPTIA variant2 in cell proliferation also in these different phenotypes. As previously observed in MCF7 a strong inhibition of proliferation and cell death induction was observed also in SK-BR-3 (Figure 3A_2_) and in the basal cell line MDA-MB-231 (Figure 3A_3_), confirming a potential role of this variant also in these breast cancer cells.

Annexin V and propidium iodide (PI) staining displayed early (annexin positive, PI negative) and late (annexin positive, PI positive) apoptotic events in siRNA2 transfected cells, as compared with mock-transfected control cells (Figure 3B). DNA staining was performed with HOECHST fluorescent dye, 72h after silencing. As expected, butyrate alone caused nuclear fragmentation, both in MCF-7 cells (Figure 3C) and in MCF12F control cells (Figure 3C, *right*). In contrast, siRNA_2_ silencing was mainly efficient in inducing apoptotic bodies in MCF-7 cells, whereas it was unable to trigger apoptosis in non neoplastic MCF12F cells. The treatment with HDAC-inhibitor butyrate in CPT1Av2 silenced MCF-7 cells did not improve the apoptotic effect (Figure 3C and 3D). In addition in MCF7 cell line, defective for the functionality of Caspase 3, a strong induction of Caspase 9 was observed by western blot analysis in CPT1Av2 silenced cells. As shown in Figure 3E, the activation of caspase 9 was strongly induced in siRNA_2_ treated cells. Similarly a strong induction of caspase 9 was observed in Na butyrate treated cells and in co-treated cells (siRNA_2_ and Na-butyrate). These results are in agreement with data obtained on apoptosis induction.

### Nuclear HDAC activity decreases in CPT1A variant 2-silenced MCF-7 cells

**Figure 4:**
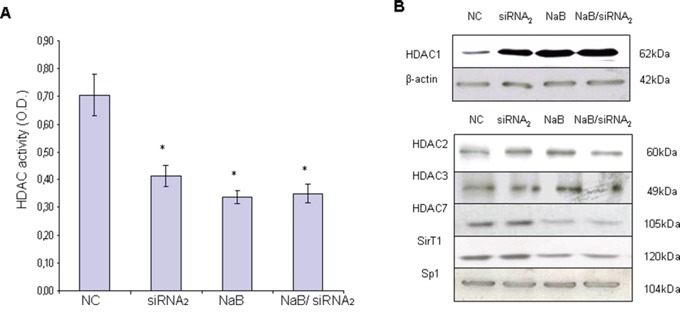
HDAC activity and HDAC1 expression in protein extracts from CPT1Av2 silenced (siRNA2) or/and treated with 5mM sodium-butyrate (NaB) MCF-7 cells **A.** Colorimetric assay was performed to analyze HDAC activity on nuclear extracts of MCF-7 cells. Histograms show the optical densities (O.D.) (means ± standard deviations) obtained by ELISA plate reader at 405 nm from three independent experiments. *P≤0.03, siRNA_2_ or butyrate-treated *versus* mock transfected cells (NC). ^#^*P*=0.1, siRNA2 and butyrate co-treated *versus* single treatments. **B.** Western blot analysis for the different classes (I, II and III) of HDAC proteins was performed on protein extracts from CPT1Av2-silenced (siRNA_2_) and/or butyrate-treated (NaB) MCF-7 cells. β-actin or Sp1 levels are reported as normalizer of the extracts.

The knock down of CPT1 variant 2 induced a significant decrease in nuclear HDAC activity (*P*= 0.03) (Figure 4A).

The protein expression of HDAC-1 in whole cell extracts inversely correlated with the HDAC activity levels (Figure 4B). In fact, in the CPT1Av2 silenced MCF-7 cells where the HDAC activity was lower, the protein expression of HDAC-1 was increased as compared with the controls. The treatment of siRNA_2_-transfected MCF-7 cells with Na-butyrate did not further affect HDAC-1 expression, as shown for the HDAC activity (Figure 4A, 4B). Furthermore, to investigate if the lowered HDAC activity was due to a decreased level of other HDAC proteins, we tested other class I HDACs molecules (HDAC2 and HDAC3), classII (HDAC7) in the nuclear extracts of silenced MCF-7 cells. Western blot analysis showed that the decrease of nuclear HDAC activity triggered by CPT1A silencing did not correlate with a corresponding decrease of HDACs class1 and class II proteins (Figure 4B). Actually no evident modulation of other HDAC molecules was induced by CPT1A silencing (Figure 4B). In order to define if CPT1-Av2 silencing could affects the expression of deacetylases of class III, we analysed the expression of SirT1 that could modifies histones through deacetylation and it is involved in tumorigenesis. As shown in Figure 4B SirT1 expression is not evidently modulated after silencing as compared to the control. Conversely Na butyrate strongly down modulate Sirt1 expression (65% of decrease) equally in CPT1-Av2 silenced cells and in not silenced cells as compared to control, suggesting that siRNAv2 do not interfere with Na butyrate modulation of Sirt1 expression. Results obtained could suggest that the significant increase of HDAC1 protein induced by CPT1A silencing (Figure 4B) could be due to a compensatory effect of the cell translational machinery, attempting to rescue the lowered HDAC activity.

### CPT1A isoform 2 interacts with HDAC1

**Figure 5:**
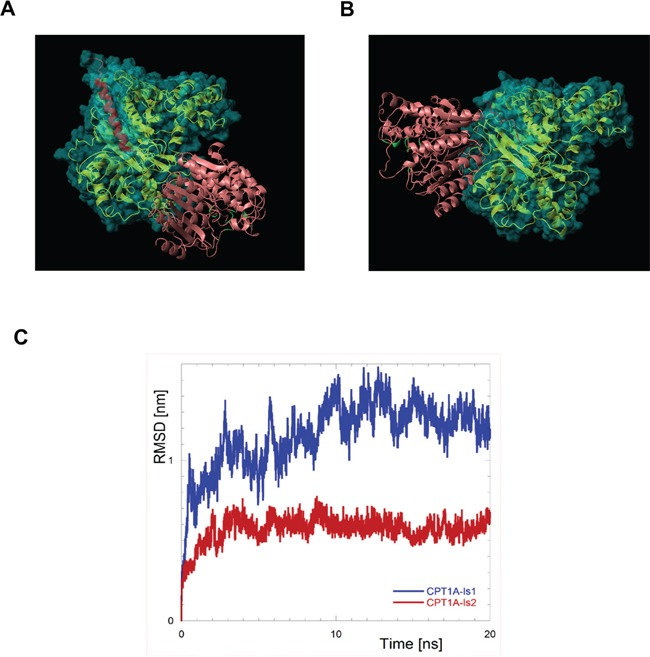
The molecular complex CPT1-Is2/HDAC1 displays an higher stability compared with the other complex formed with CPT1-Is1 **A.** Resulting complexes of CPT1-Is1/HDAC and CPT1-Is2/HDAC **B.** as obtained by molecular docking conducted by PatchDock and refined by FireDock. The CPT1 proteins are reported as gold ribbon, the residues that are different in the two isoforms (residues 746-772 for CPT1-Is1 and 746-755 for CPT1-Is2) are red colored, the protein surface is also reported as semitransparent and cyan colored. HDAC-like protein (1c3r) is reported as coral ribbon; the active site, identified as the residues in contact with the substrate [20], are depicted in green **C.** Root mean square deviations (RMSD) of CPT1A from its starting position in the complex with HDAC1. Rototranslational motions were removed by fitting the positions of HDAC atoms to their coordinates in the initial structure.

We previously had shown the CPT1A-HDAC1 nuclear interaction by immunoprecipitation and immunoblotting analyses [5]. Docking simulations were performed to confirm and characterize the molecular interface between these two molecules (Figure 5A, 5B). The structures of the human CPT1, isoform 1 and 2 (CPT1Av1 and CPT1Av2, respectively) are not available in the Protein Data Bank (http://www.rcsb.org) [27] and have been obtained by homology model. The two proteins share the great part of the sequence (773 aminoacids for CPT1Av1 and 756 for CPT1Av2) but they differ in the C-terms, starting from the position 746. In both cases the C-terms have been almost fully modeled; in the case of isoform 1 the region starting from the 746 position has been modeled essentially as an amphiphilic helix, correctly oriented. In the case of isoform 2 this region has been modeled as coiled with an amphiphilic fashion; in this conformation an underlying extended hydrophobic region is protected by an unfavorable solvent exposure. The docking has been performed by using the program PatchDock and the program Fire-Dock has been used to refine the docked models. Both of these programs were tested in CAPRI (Critical Assessment of Predicted Interactions) blind trials and were shown to give accurate results [28]. The best scoring structures for the CPT1A-Is1/HDAC and CPT1A-Is2/HDAC complexes are reported in Figure 5A, 5B. Both these structures show low values for the Firedock Global Energy Function, predicting that both CPT1Av1 or CPT1Av2 are able to interact with HDAC. Interestingly, this value is definitely more negative in the case of Isoform 2 respect to the Isoform 1 (−68 in the former case against −20 in the last case).

To verify the stability of the obtained structures we performed MD simulations on the above mentioned complexes CPT1Av1/HDAC and CPT1Av2/HDAC (Figure 5C). The root mean square deviations (RMSD) respect to the starting structures, after removal of the rototranslational motions of the proteins, were separately evaluated for each proteins in both the simulations to estimate the intrinsic stability of the protein structures during the simulations. In all the cases the RMSD remains lower than 0.4 nm, indicating that the starting structures of the proteins are retained. This evidence is particularly important in the case of CPT1Av1 and CPT1Av2, confirming the reliability of the used models, obtained by homology, as above described. To test the stability of the two complexes we have calculated the RMSD by using a different approach [29, 30, 31]. To put in evidence the inter-proteins motion we calculated the RMSD of the CPT1A, but rototranslational motions were removed by fitting the positions of the HDAC proteins. The results reported in Figure 5C clearly show a lower propensity to change the proteins arrangement respect to the starting structure in the case of CPT1Av2/HDAC, with respect to CPT1Av1/HDAC. Furthermore, also lower fluctuations of the RMSD value is observed for the CPT1Av2/HDAC complex. Both these results suggest a higher stability of the identified structure for the complex between CPT1Av2 and HDAC, respect to those obtained in the case of CPT1Av1/HDAC.

**Figure 6:**
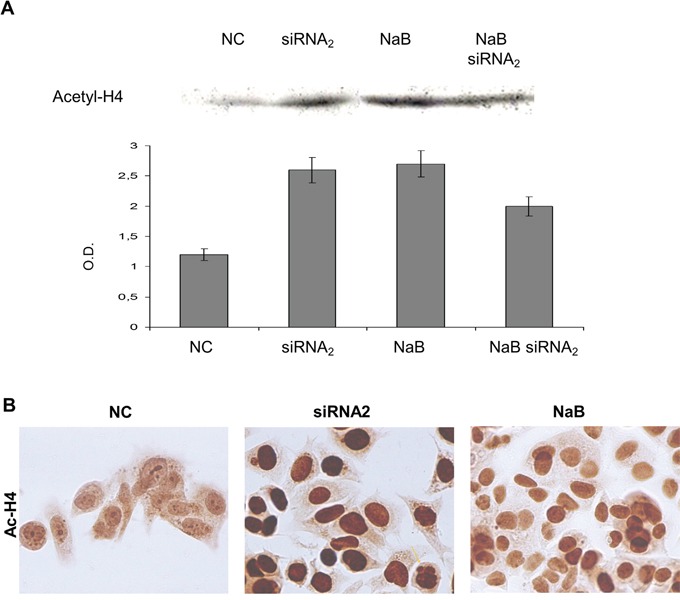
Acetyl-histone H4 expression levels in protein extracts from CPT1Av2 silenced (siRNA2) or/and treated with 5mM sodium-butyrate (NaB) MCF-7 cells **A.** Western blot analysis of acetyl histone H4 protein was performed in acid protein extracts from CPT1A2 silenced (48h) and/or butyrate treated (24h) MCF-7 cells. Optical densities (O.D.) indicate mean values ± standard deviations of three independent experiments as described in materials and methods.* *P* ≤0.02, siRNA_2_ or butyrate-treated *versus* mock transfected cells (NC). ^#^*P*=0.08, siRNA2 and butyrate co-treated *versus* single treatments. **B.** Immunostaining of acetyl histone H4 (Ac-H4) was performed on MCF-7 cells transfected for 48h with scrambled sequence (NC) or siRNAs against CPT1Av2 (siRNA2), or treated with sodium butyrate (NaB) for 24h. Apoptotic bodies were evident in siRNA2 transfected cells (arrow).

Analyzing these complexes, the CPT1A residues at the interface with HDAC, for the two complexes, are different. In the case of isoform 2 the interaction with HDAC involves a region close to the C-terminus, where the differences with CPT1Av1 are located. In particular, the residues PRO-752, SER-753, SER-754, ASP-755 and THR 756 of CPT1Av2 are in tight contact (distance lower than 5Å) with HDAC in the final structure of MD simulations. Apart the best scoring structure reported in the Figure 6, in 8 of the 10 complexes obtained after FireDock refinement the interaction with HDAC involves this region of CPT1Av2. In the case of CPT1Av1, where this region is different, stable similar complexes are not detected and the interaction with HDAC involves different protein region. Considering the great similarity of the other region of CPT1Av1 and CPT1Av2, this evidence suggests a higher stability of the complex formed by CPT1Av2 and HDAC1, further supported by the more negative value of the Firedock Global Energy Function. Finally, in both models the HDAC active site results solvent exposed, suggesting that HDAC1 activity could be retained after complexation.

### CPT1A silencing affects histone acetylation level

Western blot analysis was performed on acid soluble protein extracts to study the modulation of acetylation of histone and non histone proteins induced by CPT1 gene silencing. The effect on histone acetylation level induced by the CPTIAv2 silencing was compared to modulation induced by the HDAC inhibitor butyrate. Strikingly, the CPT1Av2 siRNAs transfection modulated the histone acetylation level (Figure 6A, 6B). A 2,5 fold increase in the acetylated histone-H4 protein was evident in the extracts from CPT1Av2-silenced MCF-7 cells, compared to those from mock-transfected cells (Figure 6A) (*P*=0.02). The increase was comparable to that induced by butyrate alone. In addition, immunocytochemistry was performed on silenced MCF-7 cells to analyze the histone acetylation level in MCF-7 cells transfected for 72hrs with scrambled sequence (Cs) or siRNAs against CPT1v2 (Figure 6B) A strong increase of acetylated histone level was observed in siRNA CPT1v2 transfected cells confirming data obtained by western blot as shown in Figure 5A.

### CPT1A2 RNA interference modulates cancer-related gene expression

**Figure 7:**
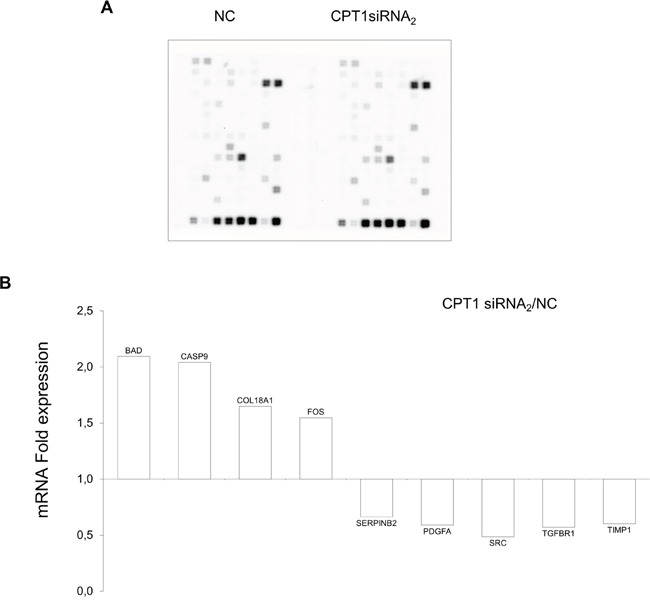
Expression analysis of CPT1Av2 silencing target genes **A.** Array image of Oligogene array cancer pathway finder performed on mRNAs extracted from CPT1A2 silenced or mock treated (NC) MCF-7 cells. **B.** The mRNA expression folds (y axis) were obtained comparing the mRNA levels detected in siRNA_2_ treated MCF-7 cells (48h) *versus* mRNA levels in mock-transfected cells (NC), as mean of triplicate experiments. Only the expression fold ≥ 1,5 was relevant and included in the histogram (*P* ≤ 0.05).

To define downstream targets of histone acetylation triggered by CPT1A silencing, a gene microarray was selected able to detect the expression of genes involved in cell-cycle control, apoptosis, invasion and metastases. Strikingly, the siRNAs targeting of the CPT1Av2 caused a significant up-regulation of the gene expression of BAD and CASP9 (Figure 7A, 7B), well-known activators of apoptosis. Moreover, we found an up-regulation of the fragment A of collagen XVIII (also known as endostatin) which has been associated with apoptotic cell death and anti-angiogenesis. On the contrary, genes such as PDGF-A, SERPINB2, TIMP1 (Figure 7B), which have been associated to poor prognosis, invasion and metastases, were down-regulated in CPT1A silenced breast cancer cells.

## DISCUSSION

Targeting the metabolic pathways of cancer is a hot topic for drug discovery. In our earlier report we identified a new isoform of carnitine palmitoyl transferase-1 present only in breast cancer cell [5].

In the present study we identified CPT1A variant 2 (CPT1Av_2_) as a new partner of HDAC expressed and localized only in the nucleus of tumoral cells, suggesting an important role in the epigenetic transcriptional regulation in cancer cells. The knock down of CPT1Av_2_ modulates histone acetylation pattern and induces apoptosis, strongly affecting cancer relevant gene expression.

Transfected cells with classical variant 1 showed an increased transferase activity, more significant in the MCF-12F cells, as compared with neoplastic MCF7. Conversely, the hyper- expression of CPT1Av_2_ in MCF7 and in MCF12F transfected cells did not affect the canonical transferase activity. In addition the CPT1Av_2_ protein in transfected cells was found in the nucleus only in MCF7 cancerous cells, indicating that its subcellular localization, and probably its nuclear function depends not merely by the splice variant sequence.

The siRNAs targeting of CPT1Av2 isoform in MCF7 cells demonstrated, for the first time, that the nuclear CPT1A would be essential for survival of neoplastic cells. In fact, the knock-down of CPT1A nuclear expression was sufficient to induce apoptosis and proliferative inhibition in MCF7, SK-BR-3 and MDA-MB-231 breast cancer cell lines, suggesting that the role of CPT1Av_2_, expressed only in cancer cells, is independent by the breast cancer phenotype molecular portraits, proposing a potential value as new tumor specific target. The induction of programmed cell death was associated to a strong increase of caspase 9 activation, a significant decrease of HDAC activity and a consequent increase of histone acetylation. Focusing on the molecular interaction between CPT1A isoforms and HDAC1 protein by docking models and MD simulations, we show that both the splice variants could interact with HDAC1 but with different binding affinity, being more stable the complex formed with the isoform 2. These “in silico” evidences are supported by silencing studies. Furthermore, an higher affinity as compared with the classical isoform 1, has been detected in the case of the isoform 2, electively expressed in cancer cells. The model obtained by docking and MD simulations for the CPT1Av2/HDAC complex is in agreement with the observed preservation of the HDAC activity, remaining the HDAC active site solvent exposed.

After silencing, genes related to cell death and good prognosis were up-regulated at transcript level. In particular, in silenced MCF-7 cells we found a higher levels of well-known pro-apoptotic factors such as BAD and Caspase-9 [32, 33] and of Col18, that acts as an endogenous inhibitor of tumor growth and angiogenesis [34]. Conversely, by gene array studies, a decreased expression was shown after CPT1A silencing of several factors, such as SERPINB2 and PDGF-A, related to cell survival and tumor progression. In particular, SerpinB2 (plasminogen activator inhibitor type 2, PAI-2) has been recently identified as a cell survival factor that binds tumor suppressor retinoblastoma protein (Rb) and protects Rb from calpain cleavage, thus enhancing cell survival [35]. Overexpression of PDGF-A (platelet-derived growth factor, alpha polypeptide) and its receptor (PDGFR-A) are associated with tumour progression in breast cancer [36]. Also TIMP metallopeptidase inhibitor-1 (TIMP-1) gene was down-regulated. This gene has mRNA splice variants differentially associated with prognosis in primary breast cancer, nevertheless a decreased expression of TIMP-1 protein is associated with recurrence-free and overall survival (OS) in breast cancer [37].

In this report, we also showed that the histone hyper-acetylation obtained in CPT1Av2silenced cells was comparable to that induced with butyrate treatment alone. Hence, CPT1Av2 gene silencing could directly induce HDAC inhibition, without requiring the association with another known HDACI.

The co precipitation with HDAC we previously published confirm the physical interaction by CPT1-Av2 and HDAC, suggesting a potential role of CPT1Av2 as co-factor. In fact data demonstrated that its presence augments HDAC activity and its depletion do not abolish completely but severely decreased its function. It is worth of note that we have seen no effects following siRNA_2_ treatment in MCF12F cells, a non cancerous mammary gland cell line which did not express detectable level CPT1Av2 in the nucleus [5]. Conversely, the HDACI butyrate showed a growth inhibitory effect on control mammary epithelial cells, confirming previous reports [38]. Data obtained point out the preferential production of CPT1Av2 in cancerous cells, suggesting that metabolic changes (such as increase of fatty acids synthesis) in neoplastic cells, that strongly are required for cancer clinical emerging and progression [39], could affect not only the mitochondrial function of CPT1A but also the balance of CPT1A isoforms production. In addition the “intracellular milieu” could drive also the localization and the nuclear function of CPT1Av2. In fact the overexpression of CPT1Av2 in MCF12 cells results not a sufficient condition to induce its the nuclear localization in this cell type. It is north of note that more aggressive phenotypes analyzed, as compared to MCF7 (derived from luminal A phenotype), such as SKBR3 and MDA-MB-231 (respectively luminal B HER2+, and basal triple negative phenotypes) express only CPT1Av2 while the CPTI-A v1, the metabolic form, is lost, suggesting a preferential splicing in favor of this variant.

As a consequence, the employment of nuclear CPT1A “cancer specific” variant 2 as a target for new anticancer therapeutics could activate cancer-relevant death genes by epigenetic regulation, in a more selective and specific manner as compared to other known HDAC inhibitors, representing an attractive and concrete opportunity to improve current strategies for breast cancer therapy.

## MATERIALS AND METHODS

### Cell culture, cloning and transfection

CPT1Av1 and CPT1Av2 isoforms were cloned into pcDNA-ha. We performed transient transfections in MCF-7 breast cancer cells (HTB-22, ATCC) and MCF12F non cancerous mammary cells (CRL-10783, ATCC), MDA-MB-231, (HBT26, ATCC), SKBR3 (HBT30, ATCC) purchased from American Type Culture Collection (ATCC) by using Calcium Phosphate Transfection Kit (Invitrogen). Cells were seeded at a density 25,000 cell/cm^2^ and grown in complete culture medium, according to condition suggested from ATCC. After an overnight culture, MCF7 and MCF12 cells were transfected with empty plasmid vector (as control) or CPT1Av1 and CPT1Av2 plasmid vectors. Glycerol was added to cultures after following 24h. The cells were harvested 72-hours after transfection.

### SiRNA transfection

Double-strand purified RNAs, 21nt in length, were pre-designed from Ambion. (Ambion Inc., USA). Three siRNAs sequences matching the CPT1Av1 (siRNA_1_), or three siRNAs targeting the CPT1Av2 (siRNA_2_) (Table 1) were used to silence the gene expression of CPT1Av1 or CPT1Av2, respectively. To silence the expression of both CPT1A isoforms, we used a mix of three sequences, two against the variant 1 and a third matching the variant 2 (siRNA_all_). A 19bp scrambled siRNA sequence that did not target any endogenous transcript was used as negative control (NC) to verify the absence of non specific effects on gene expression (Ambion). We performed transient transfections in MCF-7, in MDA-MB-231, SKBR3 cancer cells and in MCF12F non cancerous mammary cells (ATCC) grown in complete culture medium, according to condition suggested from ATCC. Cells were seeded in 6 well multi-wells at 25,000 cell/cm^2^. After an overnight culture, cells were transfected with siRNAs diluted in lipofectamine transfectant agent (Invitrogen) for 24h, 48h or 72h (120 and/or 70 nM final concentration), mixed as reported above. For co-treatment experiments, siRNAs-transfected cells were maintained 24h (48h for apoptotic assay) in culture and next sodium butyrate (Sigma-Aldrich S.r.l., I-20151 MI, Italy) was added at 5mM for other 24h (48h total incubation time, 72h for apoptotic assay).

**Table 1:** siRNA sequences designed against CPT1A transcripts and used in the study

Gene ID	Accession n.	siRNA sequence (sense)^1^*	Code
		5′-GGGUAAACUUUUGUUUUGUtt-3′	811
CPT1A variant 2	NM_001031847	5′-GGCACCUGAAAGAAGCAAUtt-3′	812
		5′-UCGGCUUGGAUUUAUUAAGtt-3′	813
		5′-AGUUCAGAUACUUGAGACAtt-3′	814
CPT1A variant 1	NM_001876	5′-UUUUCACUUGCUGUGCAUUtt-3′	815
		5′-UGUGCUGGGCUGGAAAGAAtt-3′	816

* Each RNA oligo was synthesized with a corresponding anti-sense strand (not reported) to be used into the cells (see Mat. and Methods).

### Immunocytochemistry

MCF-7 and MCF12F cells untreated, or transfected with CPT1Av1- or CPT1Av2- pcDNA-ha or alternatively with siRNA (120 nM) as reported above, were plated in 4 wells/chamberslides at a concentration of 25,000 cells/cm^2^. After an over-night culture, were fixed in formalin 10%. The primary antibodies used were anti-CPT1Av1 (H95-Santa Cruz), mouse monoclonal anti-Ha (Covance) and anti-acetyl histone H4 (Upstate). To assess the background staining, a negative control was carried out without addition of primary antibody. Secondary antibody (biotinylated goat anti-rabbit IgG) and following reagents (HRP-conjugated streptoavidin) were added. After washing, slides were incubated with diaminobenzidine (DAB) and counterstained with haematoxylin.

### RNA extraction and Real time RT-PCR

Total cellular RNA was extracted by Tri Reagent (Ambion, Inc.), according to the manufacturer's instructions. RNA quantification was performed using spectrophotometry. One microgram of RNA was used for reverse transcription using GeneAmp RNA PCR Kit (Applied BioSystems) and Random Examers as primers to cDNA synthesis. The primers used for CPT1Av1 and CPT1Av2 amplification reactions are reported in Mazzarelli *et al*., 2007. All reactions were processed in MyiQ Single-Color Real-Time PCR Detection System (Bio Rad) and results were analyzed by IQ5 program (Bio Rad). To normalize template input, β2-microglobulin (endogenous control) transcript was amplified for each sample.

### Histone deacetylase activity

Nuclear protein extracts obtained from MCF-7 cells according to the protocol previously reported [5] were used to detect HDAC activity with a colorimetric assay kit (BioVision Research Products Mountain View, CA 94043 USA). Samples were analyzed using an ELISA plate reader at 405 nm.

### CPT1 activity assay

Spectrophotometrical method was applied, using general tiol DTNB reagent in nuclear and cytoplasmic extracts from CPT1Av1 and CPT1Av2 transfected MCF-7 and MCF-12F cells.

### Western blot

Whole cell extracts or nuclear extracts were obtained as reported in Mazzarelli et al. 2007. Acid soluble proteins were extracted according to the protocol for histone proteins (UpState). Total and acid proteins extracted as above (10μg) were equally loaded (Ponceau S-staining) on 10% SDS- Proteins were transferred to a PVDF membrane (Hybond P, Amersham GE Healthcare). Primary antibodies: anti-CPT1A (Santa Cruz, H95), anti-haemoagglutinine mouse monoclonal antibody (Covance), anti-acetyl-histone H4 (Upstate) rabbit polyclonal antibodies, anti-HDAC1 (UpState, clone 2E10) mouse monoclonal antibodies, anti-HDAC2 /HDAC3 /HDAC7 (Cell Signaling Thecnology, Antibody Sampler Kit) rabbit polyclonal antibodies, anti-SirT1 (Cell Signaling Thecnology, C14H4) rabbit monoclonal antibodies and anti-Cleaved Caspase9 (Cell Signaling Thecnology, Asp330) rabbit polyclonal antibody were used. Filters were reprobed with anti-β-actin mouse monoclonal antibodies (Sigma-Aldrich, 63103 USA) or anti-Sp1 mouse monoclonal antibodies (Santa Cruz biotechnologies, Inc) to normalize respectively cytoplasmic or nuclear protein levels. Filters were developed using an enhanced chemiluminescence system (ECL, Amersham-GE Healthcare).

### Gene expression analysis by oligo gene microarray

The expression of 113 genes was analyzed by Human Cancer Pathway Finder oligogene array (EHS033, SABioscience), in MCF-7 cells transfected with CPT1Av2 siRNAs (siRNA_2_) for 48h. Cells transfected with scrambled siRNA (Cs) and untransfected cells were used as controls. Total RNA was extracted as described above, quantified by spectrophotometry and checked on agarose gel for quality. Only values showing gene up-regulation or down-regulation by 1.5 fold or more (ratio between siRNA_2_-silenced cells and mock-transfected cells mRNA levels) were reported in the study as statistically relevant (see statistical analysis section).

### Annexin V and propidium iodide staining

The MCF-7 cells seeded in 4 wells chamber slides and transfected for 72h with scrambled sequence and CPT1A siRNA2 were incubated 5 min at the dark with annexin V-FITC and propidium iodide according to condition reported by manufacturer (BioVision). Analysis was performed using a Nikon E600 fluorescence microscope.

### In silico docking analysis

Homology molecular models were generated for Carnitine Palmitoyltransferase CPT1A, isoform 1 and 2 (CPT1Av1 and CPT1Av2) with two different approaches, by using ModWeb [15] and SwissModel [16]. In the case of ModWeb, three different models have been obtained for CPT-Is1, using as templates the structures with PDB code 1ndb, 2deb and 2fy2, and two models for CPT-Is2, using 1ndb and 1t1u as templates. In the case of SwissModel the project mode approach has been used, combining the SWISS-Model pipeline tools and the program Deep-View [17] as described in [18]. For both isoforms, the used templates have been the structures with PDB codes 1t7z (reference template), 1 nm8, 1t1u, 1xml and 2h4t. Due to the good sequence similarity between the modeled sequences and the used templates, the obtained models result similar to each other and, generally, they show good evaluation parameters (like the E-values). To individuate the final models for docking simulations, we have performed a deep inspection of the C-terms regions, where the differences between the two sequences are located, excluding all the models missing this region. Between the different models, we have selected, for the two isoforms, those obtained by using the ModWeb approach, with the protein Carnitine Acetyltransferase (PDB code 1ndb) as template. The domain at the N-terminus was missed in all the obtained models. This region has the same sequence in the two isoforms and it is predicted to constitute the membrane domain. For this reason its absence represent a slight limitation in the docking simulation. The obtained structure for CPT1-Is1 defines the coordinates for the residues between 166 and 772, that correspond to the region 30-625 in 1ndb. The sequence identity between CPT-Is1 and template is equal to 31% (identity greater than 30% are considered safety), the Model score, as obtained in Modweb, has been 1.00 (value greater than 0.7 are deemed good) and the E-value has been lower than 0.0001. Analogously, for CPT-Is2, we have obtained a model for the region from the position 166 to 755. In this case, the sequence identity has been equal to 30%, the Model score has been equal to 1.00 and the E-value has been lower than 0.0001. As model for HDAC-class I protein the structure of the HDAC-like protein (PDB Code 1c3r) has been used, without refinement. *In silico* docking experiments were performed using PatchDock [19] and then further refined and re-ranked with FireDock [20]. Structural analyses and molecular graphics were performed using the program MOLMOL [21].

*Molecular dynamics (MD) simulations* of the complexes between both the CPT1A isoform (Is-1 and Is-2) and HDAC, as obtained by docking simulations, were performed using GROMACS 4.0, with the GROMOS 43a1 force field [22]. The simulations were performed according to previously described procedures [23], except for some details. In particular, after energy minimization in *vacuo*, the protein was centered in a rhombic dodecahedron box (roughly 13.2 nm wide), hydrated with roughly 49000 water molecules and an opportune amount of Na^+^ and Cl^−^ to assure electroneutrality and a salt concentration of about 0.01M. During simulations, the system was kept at constant temperature (300 K) and pressure (1 bar) by the Berendsen weak-coupling method [24]. Electrostatic interactions were calculated by the use of the particle mesh Ewald method [25]. The protonation state for the histidine aminoacids were obtained by means of the PDB2PQR server [26], by using the PropKa algorithm. Apart the equilibration, the MD simulations were 20ns long. RMSD were calculated according to standard definitions.

### Statistical analysis

The analyses were performed three times in independent experiments and values provided are means ± standard deviations (SD). The percent number of positively stained cells for CPT1 immunocytochemistry and the apoptotic index were calculated by two independent observers. Unpaired t-tests were used for normally distributed continuous variables to assess statistical differences (*P*≤0.05 was significant). The HDAC activity assay and gene arrays were performed in triplicate and the averaged values were used for statistical comparison. The gene array data sets were corrected using minimum value for background subtraction and Interquartile normalization after background subtraction.
